# Supplementary material for: Evaluation of Age-Dependent Changes in the Coloration of Male Killifish Nothobranchius Guentheri Using New Photoprocessing Methods
Source: Biology (Basel). 2022 Jan 27;11(2):205. doi: 10.3390/biology11020205 (PMC8869725; doi:10.3390/biology11020205)
Supplement: Supplementary file 1 [file biology-11-00205-s001.zip › biology-1542506-supplementary.pdf]

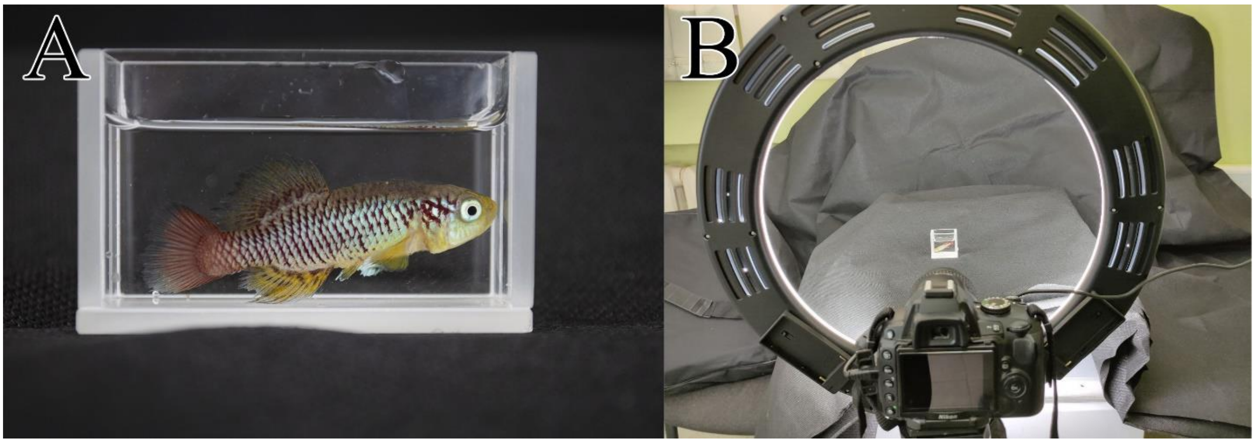

Figure S1 - Equipment for photofixation of N. guentheri. A - Aquarium tank; B - Experimental installation of the equipment with the specified lighting parameters.

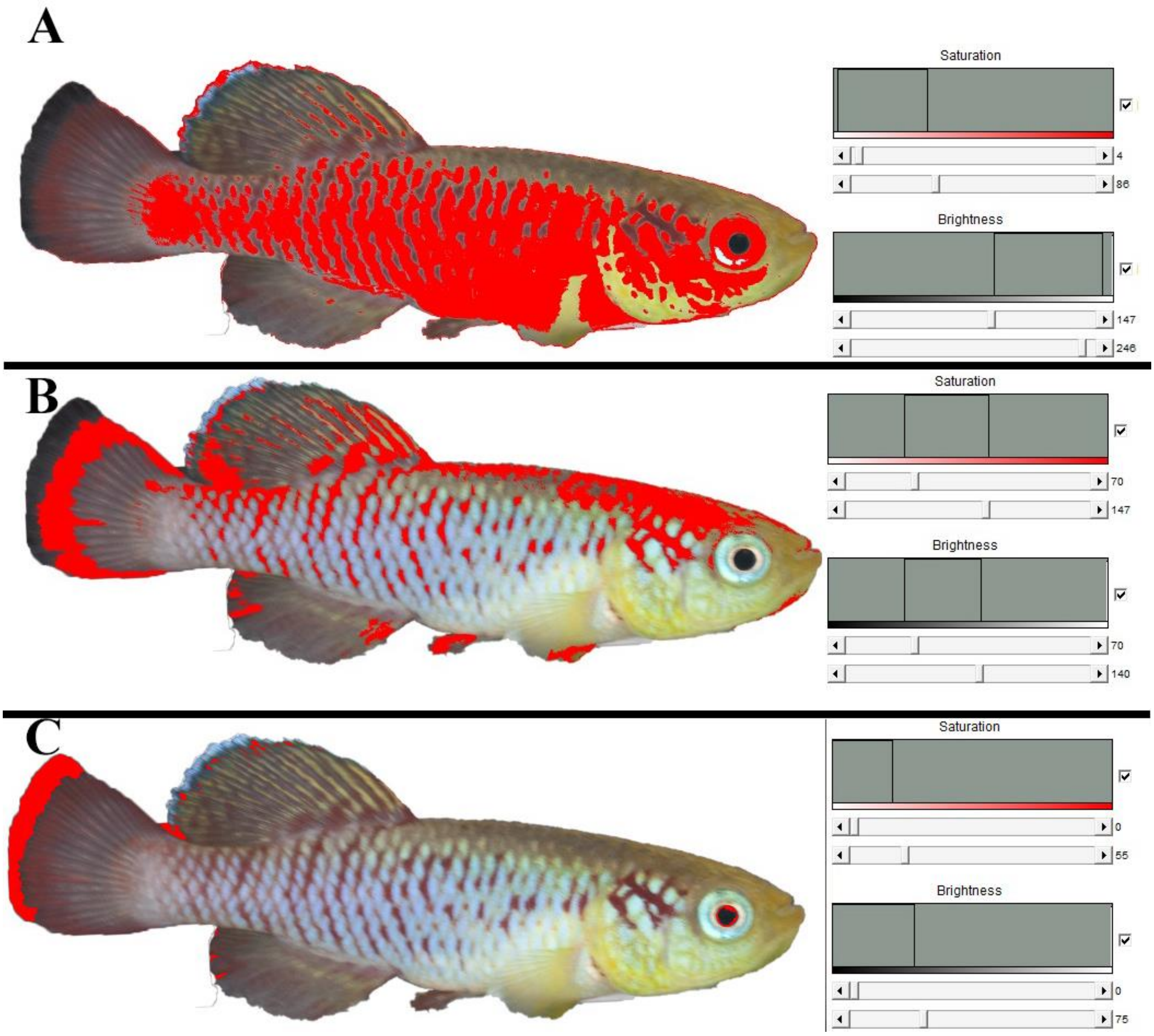

Figure S2 - Selected zones of the second group with the following values of brightness and saturation: A - LBROI, saturation - from 0±10 to 85±10; brightness - from 145±10 to 246±9. B - RROI, saturation, 70±10 to 145±5; brightness, 70±5 to 140±5; C - BROI, saturation, 0 to 55±5; brightness, 0 to 75±5. *All brightness

and saturation values (1 to 255) are applicable only to the photographs obtained in this study.

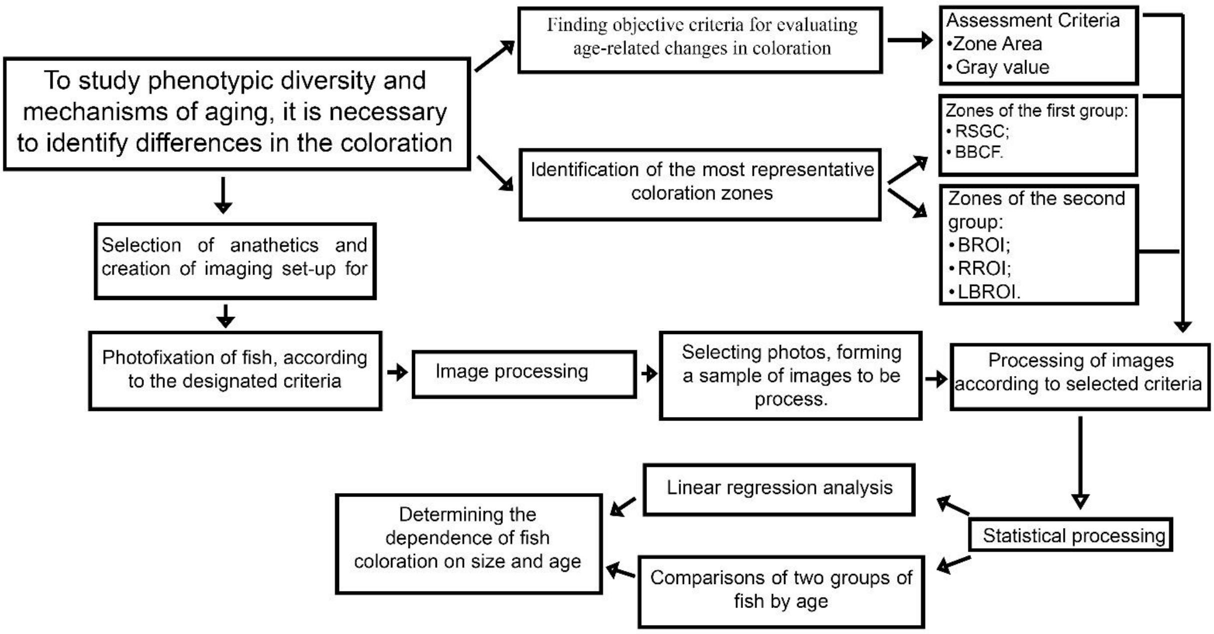

Figure S3 - Study design. The main stages of conducting and processing the material.

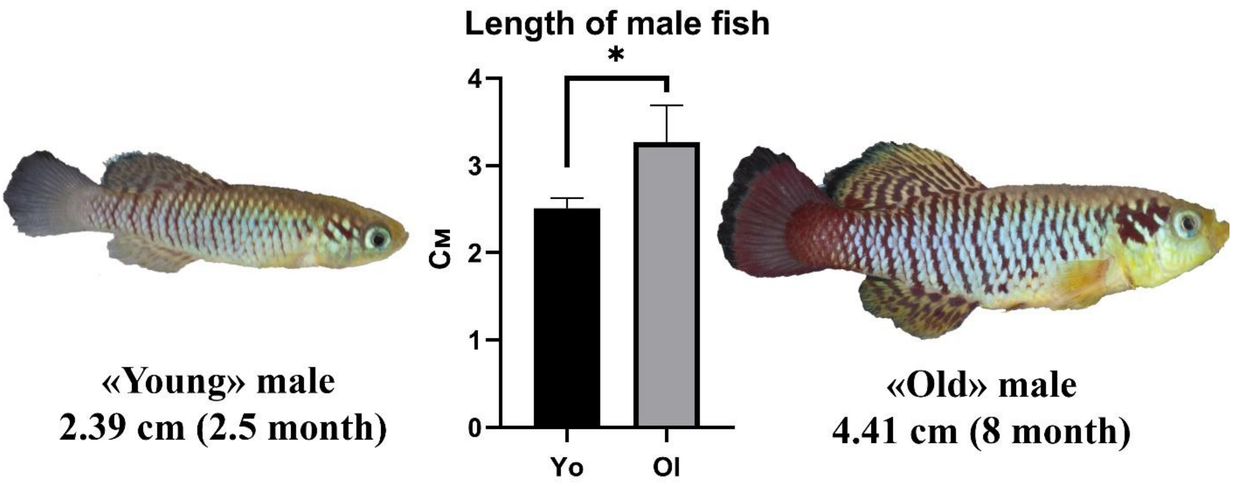

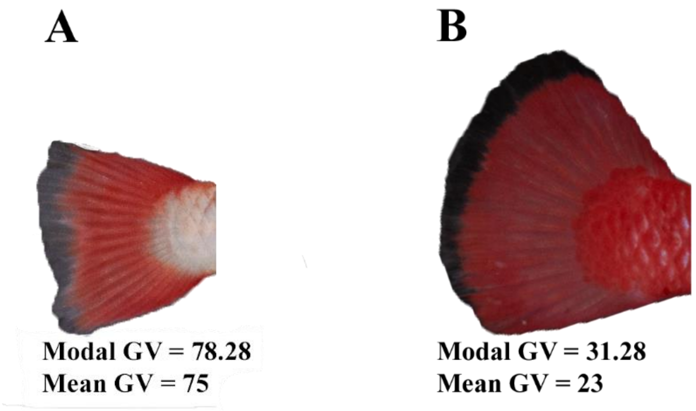

Figure S4 - Distribution of fish into groups according to size and age. (The difference in the coloration of the BBCF zone of the caudal fin of males of different age groups. A - Individual of size 2.53; Individual of size - 3.55).
